# Addressing Postpartum Care Challenges and Information Accessibility for Mothers With Hearing Disability: A Systematic Review

**DOI:** 10.7759/cureus.71092

**Published:** 2024-10-08

**Authors:** Chrysoula Rozalia Athanasiadou, Yiannis Pollalis, Athanasios Vozikis, Aikaterini Lykeridou, Vasiliki E Georgakopoulou, Panagiota Dourou, Aikaterini Sousamli, Antigoni Sarantaki

**Affiliations:** 1 Department of Midwifery, University of West Attica, Athens, GRC; 2 Department of Economics, University of Piraeus, Athens, GRC; 3 Department of Pathophysiology/Pulmonology, Laiko General Hospital, Athens, GRC

**Keywords:** deaf mothers, healthcare accessibility, healthcare professionals, hearing loss, postpartum care

## Abstract

Access to healthcare significantly impacts the health outcomes of individuals with hearing disability, underscoring the necessity for enhanced accessibility to health services. Despite this situation, there is a paucity of data regarding the provision of postpartum care for women with hearing disability. Understanding the unique experiences and needs of women with hearing disability is crucial for improving not only the health system but also the overall health of their families and, by extension, the broader community. In this systematic review, the Preferred Reporting Items for Systematic Reviews and Meta-Analyses (PRISMA) 2020 methodology was employed. Comprehensive searches were conducted across several databases, including PubMed/Medline, Scopus, BioMed Central, Cochrane Library, and Google Scholar. A total of 331 article titles and abstracts were screened based on Population/Intervention/Control/Outcome/Study/Time (PICOST) criteria, resulting in 12 articles being included in the final review. Among these, nine were qualitative studies and three were quantitative, which were reviewed using the Caldwell framework. The findings from this systematic review indicate that mothers with hearing disability often face significant inadequacies and misunderstandings concerning the information and care they receive. These inadequacies lead to a tendency to avoid or delay seeking care, or to rely heavily on self-care practices. Additionally, it was found that healthcare professionals generally lack awareness and understanding of the specific needs of mothers with hearing disability. Future research should prioritize examining both the experiences of healthcare providers and mothers with hearing disability to develop interventions that can adequately address the needs of families with mothers with hearing disability. By doing so, the healthcare system can be better equipped to provide appropriate and effective care, ultimately improving health outcomes for these families.

## Introduction and background

The most recent epidemiological projections estimate that approximately 2.5 billion individuals will experience some degree of hearing loss by 2050, with at least 700 million - or one in 10 people - requiring hearing restoration interventions. This equates to an average prevalence of 0.1% of the total population in each country [1]. The United Nations asserts that the highest attainable standard of health is a fundamental human right. However, the inequitable healthcare provision for individuals with disabilities has garnered significant attention from the international health and development community [2,3]. As early as 2009, a study emphasized the critical need to identify and address the barriers that individuals with disabilities face in accessing health services at various levels, advocating for the integration of their needs into primary healthcare systems and the implementation of effective interventions [2].

Empirical research underscores the profound impact of healthcare access on the well-being of individuals with hearing disability, highlighting the imperative to enhance access to health services [4]. Studies have shown that women with hearing disability frequently distrust healthcare professionals and services, primarily due to fears of miscommunication [4-7]. The key areas identified for improvement include the effective dissemination of information, ensuring clear and appropriate communication, building trusting relationships with healthcare providers, and fostering a sense of control among women [4, 8-10].

A population-based cohort study revealed that women with sensory impairments (both visual and auditory) have a 14% higher adjusted relative risk of severe maternal morbidity or death compared to women without disabilities. This finding indicates the necessity for prioritizing perinatal care for women with sensory impairments to mitigate adverse outcomes [11]. Furthermore, research indicates that women with disabilities are at an increased risk of emergency room visits and hospital readmissions during the postpartum period, suggesting heightened postpartum morbidity that necessitates enhanced and extended follow-up care [11, 12].

There is a paucity of data regarding postpartum care provision for women with hearing disability. However, comprehending the experiences of women with hearing disability is crucial for meeting their healthcare needs, which in turn can improve the overall health system, the health of families, and the wider community. This systematic literature review investigates the experiences and barriers encountered by parents of a mother with hearing disability during the postpartum period, aiming to enhance women's satisfaction with postnatal care services.

## Review

Materials and methods

Study Design

The Preferred Reporting Items for Systematic Reviews and Meta-Analyses (PRISMA) 2020 methodology was employed to comprehensively review the studies, documenting each step to ensure transparency and reproducibility [13]. This methodology, which promotes transparent, complete, and accurate reporting of systematic reviews, was utilized to establish a strategic design conducive to evidence-based decision-making. Additionally, the systematic review aimed to synthesize the current state of knowledge regarding postnatal care provision for parents with a mother with hearing disability. This synthesis serves to identify future research priorities, address questions unanswerable by individual studies, highlight issues in primary research, and generate or evaluate theories on the occurrence of certain phenomena. This systematic review has been registered in the International Prospective Register of Systematic Reviews (PROSPERO) with ID number 593316. 

Search Strategies

A thorough search was conducted across several online databases, including PubMed/Medline, Scopus, BioMed Central, Cochrane Library, and Google Scholar. Multiple search terms were initially used, culminating in the selection of two algorithms. The search terms related to midwifery, health promotion, labor period, and mothers with hearing disability were incorporated, along with specific exclusion terms. Table 1 presents the algorithms used and their corresponding databases where the searches were performed.

**Table 1:** Enumeration algorithms.

Research question	Algorithm	Numeration	Database
To explore the experiences and barriers presented to parents of a mother with hearing disability, regarding the care they received during the postpartum period	(deaf-mothers OR deaf-mother OR hearing-loss-mothers OR deafness-mothers OR hearing-loss-mother OR deafness-mother OR Deaf-women OR hearing-loss-women OR deafness-women OR Deaf-woman OR hearing-loss-woman OR deafness-woman OR sensory-disabilities) AND (postnatal-care OR postnatal-support OR postnatal-services OR maternity-support OR maternity-care OR maternity-services OR midwifery-support OR midwifery-care OR midwifery-services OR postpartum-care OR postpartum-support OR postpartum-services OR Maternal-health-services OR puerperium) NOT (infant-hearing-loss OR pediatric-hearing)	1	PubMed/Medline Google Scholar BioMed
	(deaf AND mothers OR hearing AND loss AND mothers OR deafness AND mothers OR deaf AND women OR hearing AND loss AND women OR deafness AND women OR deaf AND woman OR hearing AND loss AND woman OR deafness AND woman OR sensory AND disabilities OR deaf AND mother OR hearing AND loss AND mother OR deafness AND mother OR sensory AND disabilities) AND (postnatal AND care OR postnatal AND support OR postnatal AND services OR maternity AND support OR maternity AND care OR maternity AND services OR midwifery AND support OR midwifery AND care OR midwifery AND services OR postpartum AND care OR postpartum AND support OR postpartum AND services OR maternal AND health AND services OR puerperium) AND NOT (infant AND hearing AND loss OR pediatric AND hearing)	2	Scopus Cochrane Library

Inclusion and Exclusion Criteria of Studies

The eligibility criteria for the articles were defined using the acronym PICOST, applied as follows: (a) Population: Studies included in the analysis focused on mothers with hearing disability or mothers who belong to the group of women with sensory disabilities; (b) Intervention: The studies examined the provision of postpartum care to parents with a mother with hearing disability; (C) Comparison: The studies compared postpartum care provided to families with a mother with hearing disability or a mother with sensory disabilities; (D) Outcome: The included studies presented the experiences and barriers faced by parents of a mother with hearing disability concerning the care received during the postpartum period; (E) Study: The inclusion criteria were limited to primary research, encompassing both quantitative and qualitative studies. The excluded were studies not accessible in full text or written in languages other than English and (F) Timely: Studies published between January 1, 2010, and March 1, 2024, were included, while those outside this period were excluded.

Search Results and Data Extraction

Following the initial search of the online databases, each article was meticulously reviewed to identify the most suitable ones. During the first stage of selection, article titles and abstracts were scrutinized to eliminate those not directly relevant to the focus on the provision and assessment of care for mothers with hearing disability or women with sensory impairment during the puerperium period.

The following variables were extracted from eligible studies: first author, year of publication, title, journal of publication, year and country of study, participants, focus group (mother with hearing disability or mother with sensory impairment), research methodology, and the themes that emerged from each primary study.

The initial search was conducted in the online databases PubMed/Medline, Scopus, BioMed Central, Cochrane Library, and Google Scholar. This search yielded a total of 345 records: 37 from PubMed/Medline, 139 from Google Scholar, 27 from Scopus, seven from BioMed Central, and 135 from the Cochrane Library. After removing 15 duplicate records, 330 unique records remained for further consideration. The titles and abstracts of these 330 records were reviewed to assess their appropriateness.

Table 2 presents the number of articles retrieved from each database, the total number of articles with duplicate entries, the total number of duplicate entries, and the total number of articles without duplicate entries.

**Table 2:** Number of articles from each database, duplicate entries, and the total without duplicate entries.

Databases					Set with duplicate entries	Duplicate entries	Total without duplicate entries
PubMed/Medline	Google Scholar	Scopus	BioMed	Cochrane Library	345	15	330
37	139	27	7	135			

Following the initial search of the online databases, each article was meticulously reviewed to select the most appropriate ones. The first stage of selection involved screening the titles and abstracts to exclude articles that were not directly related to the study's focus on the provision and evaluation of care for mothers with hearing disability or mothers with sensory disabilities in the postpartum period. This process resulted in the exclusion of 298 articles that were not directly relevant, reducing the number of articles from 330 to 32.

Subsequently, an attempt was made to retrieve the full texts of these 32 articles, but four articles could not be retrieved, reducing the total number to 28. Further exclusions were made based on language and availability: five studies were written in languages other than English, three studies lacked full-text availability, and eight studies fell outside the specified time frame, reducing the number of eligible articles from 28 to nine.

The selected articles were categorized into three general groups: (a) research articles (seven articles); (b) quantitative research (two articles); (c) qualitative research (five articles); (d) bibliographic reviews (one article), and (e) articles including comments, criticisms, or suggestions for new studies (one article). Category 1 was further divided into subcategories: Category 1a included research with quantitative results, and Category 1b included research with qualitative results. One article from Category 2 and four articles from Category 3 were excluded as they did not pertain to primary research, reducing the number from 12 to seven articles. To enhance the comprehensiveness of the review, an additional five relevant articles were identified and included, resulting in a total of 12 articles.

Risk of Bias Assessment

The risk of bias in the included studies was assessed using the Caldwell framework for both qualitative and quantitative research. For quantitative studies, the Cochrane Risk of Bias tool was applied to assess selection bias, performance bias, detection bias, attrition bias, and reporting bias. Each study was categorized into low, medium, or high risk of bias based on these criteria. Qualitative studies were assessed using the Critical Appraisal Skills Programme (CASP) checklist, evaluating aspects such as research design, recruitment strategies, data collection methods, and researcher-participant relationships. 

Results

**Figure 1:**
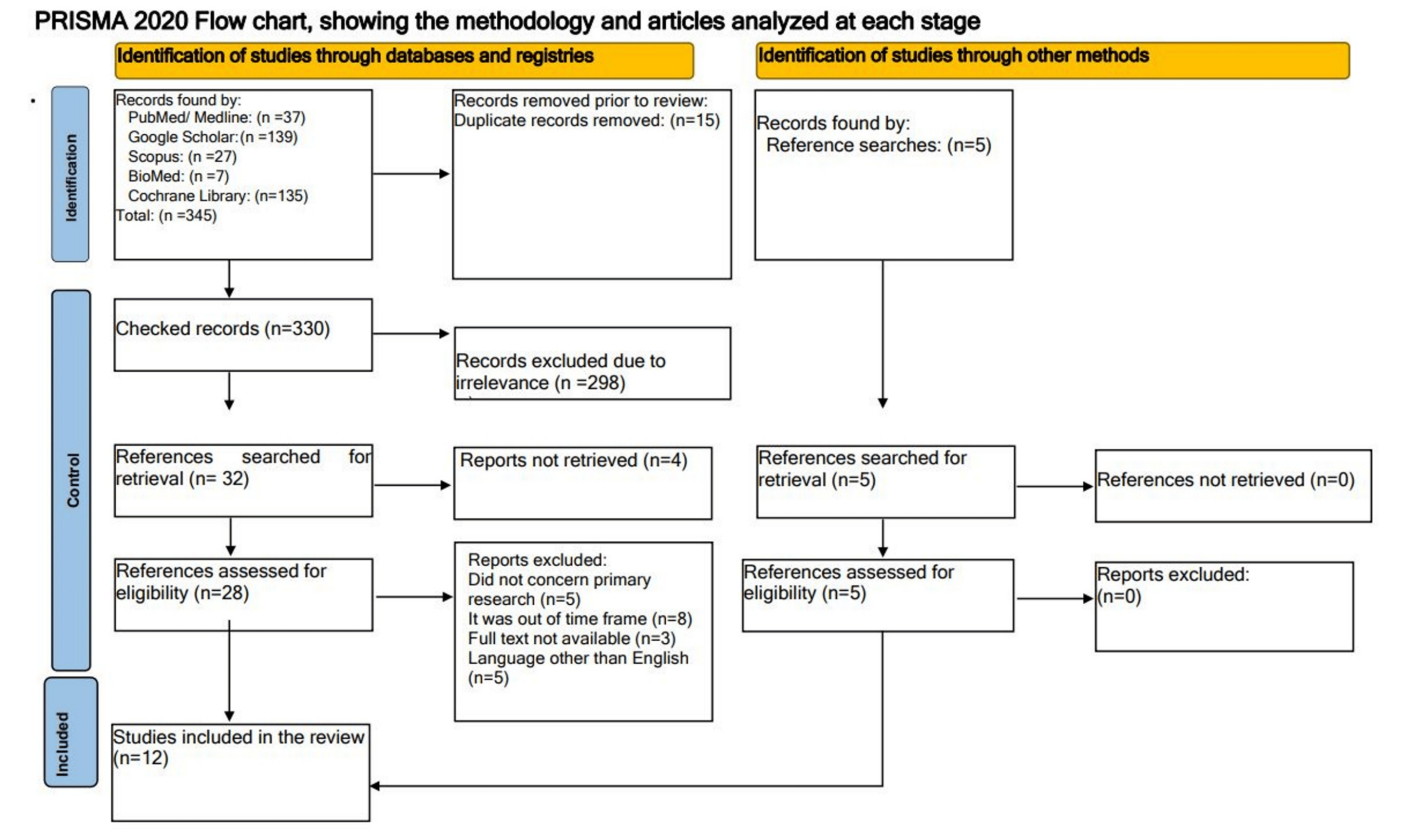
Selection process for the included studies.

The final selection comprised nine qualitative studies and three quantitative studies. The selection process is visually represented in a flowchart (Figure 1) in accordance with the PRISMA 2020 guidelines for systematic reviews.

We analyzed 12 articles published between 2010 and 2020 that focused on the experiences of mothers with hearing disability regarding their access to healthcare or maternity care. The studies were conducted in various locations: the United Kingdom (n=3), Ireland (n=1), the United States (n=2), Canada (n=2), Austria (n=1), Cape Town (n=1), Ghana (n=1), Cameroon (n=1), and Nepal (n=1).

After a thorough review of these articles, key features from each of them were entered into an Excel spreadsheet (Microsoft Corp., Redmond, WA) in ascending chronological order of publication (Table 3). This analysis revealed three main issues related to the provision of care to mothers with hearing disability during labor: (a) insufficient or wrong approach, update, and care; (b) health professionals’ lack of awareness, and (c) avoidance of care, delayed care, and self-care by the mothers with hearing disability. Table 3 summarizes the key findings from the included studies [8, 14-24].

**Table 3:** Overview of the key points of the reviewed studies.

N	First Author (Year of Publication)	Title	Journal	Country	Year the Study Was Conducted	Method	Participants	Focus Group	Insufficient or Wrong Approach, Update, and Care	Lack of Awareness of Health Professionals	Avoidance of Care, Delayed Care, and Self-Care by Mothers with Hearing Disability
1	Redshaw et al. (2013) [14]	Women with disability: The experience of maternity care during pregnancy, labour and birth and the postnatal period	BMC Pregnancy and Childbirth	England	2010	Quantitative research, secondary analysis	24,155 mothers, 1482 mothers with disabilities	197 mothers with sensory impairment	Lack of proper communication; need for more home visits; need for more help with breastfeeding	Lack of participation in decisions; less likely to be treated with kindness and understanding	-
2	Chin et al. (2013) [15]	Deaf mothers and breastfeeding: Do unique features of deaf culture and language support breastfeeding success?	*Journal of Human Lactation* (official journal of International Lactation Consultant Association)	United States	2013	Qualitative study, community-based participatory research (CBPR) approach	15 Deaf mothers who use American Sign Language	15 Deaf mothers who use American Sign Language	-	-	Strong self-care and self-advocacy skills as well as mutual support Instant information sharing with other deaf people; Using technology for social support and information
3	Schildberger et al. (2017) [16]	Experiences of Austrian mothers with mobility or sensory impairments during pregnancy, childbirth and the puerperium: a qualitative study	BMC Pregnancy and Childbirth	Austria	2013-2015	Qualitative study with semi-structured interviews	10 mothers participated in the study; 4 with a physical disability and 6 with a sensory disability (4 blind and 2 deaf mothers)	2 Deaf mothers	Sufficient information was missing regarding the puerperium; they received little or insufficient support and assistance; need for more detailed information on the actual situation and on the required interventions	Environment of discriminatory attitude; lack of support; lack of confidence in their parenting abilities, which negatively affected their self-efficacy and self-awareness; violations of personal boundaries - feeling watched and controlled; communication with health professionals was often characterized by mutual aspects of fear, uncertainty, and awkwardness	-
4	Malouf et al. (2017) [8]	Access and quality of maternity care for disabled women during pregnancy, birth and the postnatal period in England: data from a national survey	BMJ Open	England	2015	Quantitative research, secondary analysis	20,094 mothers, 1958 mothers with disabilities	174 mothers with sensory impairment	Providing information in an inappropriate format - their personal circumstances were not taken into account; there was no effective dissemination of information; appropriate communication not ensured; lack of direct communication; they wanted more postpartum contact and information about their physical recovery after birth; help with infant feeding	Lack of understanding; ;ack of kindness; partner exclusion; lack of supporting women's sense of control; lack of trusting relationships with health care providers; lack of animation	-
5	Gichane et al. (2017) [17]	"They must understand we are people": Pregnancy and maternity service use among signing Deaf women in Cape Town	Disability and Health Journal	Cape Town	2015	Qualitative study, interviews	42 Deaf mothers, users of South African Sign Language	42 Deaf mothers, users of South African Sign Language	Language barrier - poor communication in maternal services; lack of interpretation services; lack of information on infant nutrition; lack of information on postpartum care	Lack of understanding of deafness - cultural barrier Mistreatment Fear or mistrust	Delay in seeking care; avoid seeking care; lost opportunities for education and information about infant feeding and parenting skills; acceptance that they will receive inferior levels of care
6	Hubbard et al. (2018) [18]	Promoting Best practice for perinatal care of deaf women	Nursing for Women's Health	United States	2018	Qualitative study, pilot, descriptive study	5 Deaf mothers	5 Deaf mothers	Bad communication; use of a mask by healthcare professionals prevents one from reading lips; Venic catheter in the dominant arm; the same interpreter or team was wanted on a long stay; immediate attendance of an interpreter; more time was needed for communication	Mistreatment by hospital staff	-
7	Saeed et al. (2022) [19]	Barriers to and facilitators of effective communication in perinatal care: a qualitative study of the experiences of birthing people with sensory, intellectual, and/or developmental disabilities	BMC Pregnancy and Childbirth	Ontario, Canada	2019-2020	Qualitative study with semi-structured interviews	17 mothers with disabilities	4 Deaf mothers	Barriers to effective communication; lack of provider policies, guidelines and knowledge about people with disabilities and their communication needs; do not have interpreters or the patients pay for them; providers refused to provide access to communication aids and services; instead of providing access to communication, participants described providers often relying on family members to facilitate communication	Lack of provider effort	-
8	Tarasoff et al. (2023) [20]	Unmet needs, limited access: A qualitative study of postpartum health care experiences of people with disabilities	Journal of Advanced Nursing	Ontario, Canada	2019-2020	Qualitative study with semi-structured interviews	31 mothers with physical, sensory, and intellectual/ developmental disabilities	4 Deaf mothers	There was an interpreter only for the birth and not afterwards to help with both physical health and recovery, as well as psychological support, breastfeeding, home support, and early childhood care support; lack of continuity of care; lack of adequate care	Lack of provider awareness; fear of judgment, discrimination, and intrusive surveillance	-
9	Ganle et al. (2016) [21]	Challenges women with disability face in accessing and using maternal healthcare services in Ghana: Q qualitative study	PLoS One	Ghana	2012-2015	Qualitative study with semi-structured interviews	72 women with physical, visual, speech, and hearing impairments	12 Deaf mothers	Communication problems; unfriendly healthcare infrastructure	Healthcare providers’ insensitivity and lack of knowledge	Mobility problems; limited support
10	Hall et al. (2018) [22]	Dignity and respect during pregnancy and childbirth: A survey of the experience of disabled women	*BMC Pregnancy and Childbirth *	United Kingdom and Ireland	2016	Quantitative and qualitative study using an internet-based survey	37 women	2 Deaf women	Lack of reasonable adjustments or accommodations; lack of continuity of care; inadequate physical environments, space, and equipment	Maternity care providers' lack of awareness and attitudes towards disability; lack of knowledge about disability's impact on pregnancy and childbirth	Less choice and control over their experience; felt treated less favourably because of their disability
11	Morrison et al. (2014) [23]	Disabled women's maternal and newborn health care in rural Nepal: A qualitative study	*Midwifery *	Nepal	2013	Qualitative study using semi-structured interviews	27 women	10 Deaf women	Lack of reasonable adjustments; inadequate physical environments, space, and equipment	Lack of experience and knowledge among providers about disabled women's needs; health workers felt unprepared to meet the maternal health needs of disabled women	Fear of bringing shame on the family; preferred to deliver at home due to embarrassment and economic barriers
12	DeBeaudrap et al. (2019) [24]	Disability and access to sexual and reproductive health services in Cameroon: A mediation analysis of the role of socioeconomic factors	International Journal of Environmental Research and Public Health	Cameroon	2015	Quantitative study, cross-sectional survey	310 women	Women with difficulty in hearing, using hearing aid, or are deaf	More difficulties in accessing sexual and reproductive (SRH) services compared to non-disabled peers; lower satisfaction with SRH services	Lack of socioeconomic support; limited access to education and work opportunities	Restricted access to SRH services; poor utilization of family planning and HIV testing services

The following sections summarize the main findings from the analysis of the articles within each thematic area.

Inadequate or incorrect approach, information, and care

Inadequacies in Information and Care for Mothers With Hearing Disability

The analysis reveals significant inadequacies in the information and care received by mothers with hearing disability, particularly concerning communication. A fundamental issue is the lack of policies, guidelines, experience, and knowledge among healthcare providers regarding the needs of people with disabilities, specifically their communication requirements [19]. The findings highlight insufficient support and inadequate information provided on various critical aspects, including the puerperium [16, 17, 20], infant nutrition [17], and physical recovery post-birth [8]. There is a clear necessity for more detailed and accurate information about the actual circumstances and required interventions in each process [16].

Moreover, it was evident that more time needs to be dedicated to families of a mother with hearing disability to enhance communication [18]. Reports indicate a need for increased home visits [8, 14] and additional assistance with breastfeeding [8, 14].

Communication Barriers

The analysis identified significant issues with the communication approach. Mothers with hearing disability frequently encountered inappropriate and indirect communication methods that failed to consider their personal circumstances, leading to ineffective information dissemination [8, 14, 17, 18, 19]. For instance, the use of masks by health professionals impeded lip-reading, and the placement of intravenous catheters in the dominant hand hindered sign language communication [18].

Interpretation Services

There is a notable deficiency in the provision of interpretation services [17], with some cases reporting a refusal to provide such services due to cost, or the costs being transferred to the patient [19]. Often, providers relied on family members of mothers with hearing disability to facilitate communication rather than providing professional interpretation services [19]. When available, it is crucial to ensure the presence of a sign language interpreter as soon as a woman arrives at the health facility and to maintain consistency by using the same interpreter or group of interpreters throughout a long stay to foster trust and security [18].

Despite the availability of interpreters during childbirth, there is often a lack of interpreters for postnatal care, which includes physical health and recovery, psychological support, breastfeeding, home support, and early pediatric care [20]. This absence contributes to a lack of continuity of care. Research in Ghana revealed that healthcare workers exhibited insensitivity and insufficient understanding of the specific maternity care requirements of women with disabilities, resulting in inadequate services that failed to meet their needs [21].

Health Professionals' Lack of Awareness

The care received by mothers with hearing disability is significantly impacted by healthcare professionals' lack of understanding of deafness, creating a substantial cultural barrier between mothers with hearing disability and healthcare services [17]. This care is often marked by a lack of awareness, support, empathy, kindness, and understanding within an environment of discriminatory attitudes and practices [8, 16, 20]. Furthermore, studies have described the care provided to mothers with hearing disability as maltreatment [17, 18].

Research indicates that mothers with hearing disability are frequently excluded from participating in decisions that affect them, with reports also detailing the exclusion of their partners [8, 14]. Health professionals often express a lack of confidence in the parenting skills of mothers with hearing disability and provide insufficient support to foster their sense of authority. This undermines the mothers' confidence in their abilities and their self-perception, leading to violations of personal boundaries and fostering feelings of invasive monitoring and authority [16, 20]. Women with hearing disability not only face a lack of trust but also experience a fear of being judged [20].

According to Hall et al. [22], a majority of women reported that maternity care professionals did not have sufficient information or appropriate attitudes towards disability. This lack of awareness was evident in the failure to provide necessary modifications or accommodations, resulting in diminished autonomy and self-respect for the women. Significant concerns were also identified in areas such as communication, continuity of care, and physical settings.

Morrison et al. [23] emphasized that health professionals in rural Nepal lacked the necessary training to meet the unique needs of disabled women effectively. This lack of training resulted in inadequate care and a reluctance among disabled women to utilize healthcare services. Similarly, DeBeaudrap et al. [24] found that healthcare practitioners in Cameroon often lacked the knowledge and education required to meet the sexual and reproductive health needs of individuals with disabilities. This lack of expertise led to decreased satisfaction with sexual and reproductive health services and increased barriers to obtaining treatment.

Communication with healthcare professionals was often characterized by mutual fear, mistrust, uncertainty, and awkwardness, with a notable absence of efforts to build trusting relationships [16]. Additionally, there was a significant lack of effort for communication on the part of providers [19].

*Mothers* *With Hearing Disability Avoid Care, Delay Care, or Engage in Self-Care*

Mothers with hearing disability often avoid or delay access to care due to the lack of understanding and communication they have previously experienced. This delay in care results in missed opportunities for education on infant feeding and parenting skills training [17]. Chin et al. [15] found that while women with hearing disability use technology for social support and information, they also rely heavily on their self-care and self-advocacy skills, mutual support, and direct information sharing within the community with hearing disability.

Hall et al. [22] reported that a significant proportion of women with disabilities felt their rights were inadequately respected and that they were treated less favorably because of their disability. This led to a lack of trust in healthcare practitioners, deterring these women from seeking critical treatment. The absence of appropriate accommodations and effective communication exacerbated their reluctance to engage with healthcare services, resulting in avoidance or delays in seeking treatment.

Morrison et al. [23] found that disabled women in rural Nepal often opted for home births due to feelings of humiliation, financial constraints, and fear of mistreatment. The study revealed that many women avoided institutional care because of previous negative experiences and a perceived lack of understanding and support from healthcare staff. This avoidance perpetuates poor maternal health outcomes for disabled women.

Similarly, DeBeaudrap et al. [24] discovered that individuals with disabilities in Cameroon frequently avoided obtaining sexual and reproductive health (SRH) services due to past negative experiences and the belief that their needs would not be met. Financial constraints and a lack of proper accommodations further exacerbated this avoidance, leading to delays in receiving necessary treatment.

Discussion

This systematic review found that mothers with hearing disability experience significant inadequacies and misunderstandings regarding the information and care they receive during the postpartum period. Individuals with hearing disability often find that healthcare environments do not support their communication needs, leading to feelings of frustration and alienation. For instance, healthcare providers may lack basic knowledge of sign language, rely heavily on written communication that may not be comprehensible, or fail to provide interpreters during critical consultations. As highlighted by other studies, these communication failures contribute to a pervasive sense of distrust among patients with hearing disability [5-7].

Due to these persistent communication barriers, mothers with hearing disability frequently avoid or delay seeking necessary medical care. The hesitation to engage with healthcare services is largely driven by previous negative experiences where their needs were not adequately met [25]. This avoidance behavior not only compromises their health but also that of their newborns, as they might miss out on essential postpartum care and guidance. Additionally, when formal care is inaccessible or perceived as unhelpful, mothers with hearing disability often resort to self-care practices. While self-care can be beneficial, it is not a substitute for professional medical advice and intervention, especially in complex postpartum scenarios [26].

Furthermore, the lack of cultural competence among healthcare providers exacerbates these issues. Many healthcare professionals do not fully understand the cultural and social aspects of deafness, which can affect how they interact with patients with hearing disability and address their concerns. This cultural disconnect can lead to a paternalistic approach, where healthcare providers make decisions without fully involving the patient, thereby undermining the autonomy and confidence of mothers with hearing disability [27].

The findings underscore the necessity of establishing appropriate policies and guidelines and providing relevant training to healthcare providers. Such measures would help providers gain the necessary experience and knowledge to address the communication needs of people with disabilities. Current general perinatal care guidelines do not mention people with disabilities [28], and few perinatal care providers receive disability-related education and training [29].

The recommendations for improving postpartum care for mothers with hearing disability include several key strategies. Firstly, it is imperative to provide training to healthcare personnel in basic sign language and to ensure they are continuously informed about resources and technological advancements that enable culturally competent communication [19]. Additionally, hiring professional interpreters in healthcare facilities is essential. Ensuring that the same group of interpreters serves the same woman throughout her stay can significantly enhance communication and trust. Providing written information, allocating more time for patient interaction, employing trained personnel, and increasing postnatal visits where necessary are also critical steps [8, 17]. The utilization of technological resources and the presence of trained staff are vital in supporting individuals with sensory disabilities [19, 30].

Building trusting and respectful relationships between healthcare providers and patients is essential for supporting women's sense of control over their healthcare experiences. This requires providers to listen attentively, offer consistent support, and communicate effectively, thereby bridging gaps and delivering optimal services to families [19]. Improving these areas will help foster trust between women and the health system, preventing mothers with hearing disability from avoiding or delaying care or relying excessively on self-care. Such avoidance or delay can lead to increased morbidity and mortality rates for both mothers and their newborns, as well as the dissemination of misinformation from unreliable sources.

A study highlighted that service providers often refer women with disabilities from primary to secondary and tertiary health services based solely on their impairments, including hearing and communication challenges. The providers pointed out their limited knowledge and training about disability services and their difficulty in tailoring messages due to this lack of training. Additionally, they reported a lack of home support, as the health system could not afford to provide home visits [31]. Addressing these gaps through better training and resource allocation is crucial for improving the healthcare experience and outcomes for mothers with hearing disability.

Developing specific guidelines and policies that address the needs of mothers with hearing disability within the healthcare system is essential. These guidelines should be integrated into existing maternity care protocols to ensure that mothers with hearing disability receive equitable and comprehensive care. This includes establishing standards for the provision of interpreters and the use of accessible communication tools​​. In addition, continuous research is needed to monitor the effectiveness of implemented policies and to identify new challenges faced by mothers with hearing disability. Engaging with communities with hearing disability to understand their experiences and gather feedback can inform ongoing improvements in care practices​​.

Several limitations should be noted in this systematic review. First, the included studies were geographically diverse, but the majority were concentrated in high-income countries, limiting the generalizability of the findings to low- and middle-income settings where healthcare infrastructure and cultural perceptions of disability may differ. Second, many of the studies relied on self-reported data, which could introduce recall bias or subjective interpretations of care quality. Additionally, there were gaps in the availability of data on specific subgroups of mothers with hearing disability, such as those with co-occurring disabilities, which restricts the breadth of the findings. Moreover, the exclusion of non-English language studies could have introduced selection bias. Lastly, the heterogeneity of study designs and outcomes limited the ability to perform a meta-analysis, meaning that findings are largely qualitative and should be interpreted with caution. Finally, publication bias may be present, as studies with positive or significant findings are more likely to be published, potentially skewing the overall conclusions of the review.

## Conclusions

This study provides critical evidence regarding the experiences and barriers faced by parents of mothers with hearing disability or sensory-impaired mothers during the postnatal period. It was found that the care provided often fails to meet the specific needs of these families, leading to delays in seeking future care or complete avoidance of medical services. Such behavior significantly contributes to adverse health outcomes for both mothers with hearing disability and their newborns. To effectively address these issues, it is imperative that a strategic and well-organized plan of action is developed in the healthcare system. This plan should include the establishment of appropriate policies and guidelines tailored to optimally meet the communication needs of individuals with sensory disabilities during the perinatal period. Implementing such measures is crucial to ensure positive experiences and outcomes for mothers with hearing disability and their families.
